# Can Dietary Fatty Acids Affect the COVID-19 Infection Outcome in Vulnerable Populations?

**DOI:** 10.1128/mBio.01723-20

**Published:** 2020-07-23

**Authors:** J. C. Onishi, M. M. Häggblom, S. A. Shapses

**Affiliations:** a Department of Biochemistry and Microbiology, School of Environmental and Biological Sciences, Rutgers, The State University of New Jersey, New Brunswick, New Jersey, USA; b Department of Nutritional Sciences, School of Environmental and Biological Sciences, Rutgers, The State University of New Jersey, New Brunswick, New Jersey, USA; c New Jersey Institute for Food, Nutrition & Health, School of Environmental and Biological Sciences, Rutgers, The State University of New Jersey, New Brunswick, New Jersey, USA; d Department of Medicine, Robert Wood Johnson Medical School, Rutgers, The State University of New Jersey, New Brunswick, New Jersey, USA; University of Geneva; UC San Diego; University of California, San Francisco; Anglia Ruskin University

**Keywords:** COVID-19, cytokine storm, diet, endotoxin, gut bacteria, intestine

## Abstract

There is high mortality in coronavirus disease 2019 (COVID-19)-infected individuals with chronic inflammatory diseases, like obesity, diabetes, and hypertension. A cytokine storm in some patients after infection contributes to this mortality. In addition to lungs, the intestine is targeted during COVID-19 infection. The intestinal membrane serves as a barrier to prevent leakage of microorganisms and their products into the bloodstream; however, dietary fats can affect the gut microbiome and may increase intestinal permeability. In obese or diabetic individuals, there is an increase in the abundance of either Gram-negative bacteria in the gut or their product, endotoxin, in systemic circulation. We speculate that when the COVID-19 infection localizes in the intestine and when the permeability properties of the intestinal membrane are compromised, an inflammatory response is generated when proinflammatory endotoxin, produced by resident Gram-negative bacteria, leaks into the systemic circulation. This review discusses conditions contributing to inflammation that are triggered by microbially derived factors from the gut.

## Abstract (precis)

There is high mortality in coronavirus disease 2019 (COVID-19)-infected individuals with chronic inflammatory diseases, like obesity, diabetes, and hypertension. A cytokine storm in some patients after infection contributes to this mortality. In addition to lungs, the intestine is targeted during COVID-19 infection. The intestinal membrane serves as a barrier to prevent leakage of microorganisms and their products into the bloodstream; however, dietary fats can affect the gut microbiome and may increase intestinal permeability.

## OBSERVATION

The severe acute respiratory syndrome coronavirus 2 (SARS-CoV-2) has caused the CoV disease 2019 (COVID-19) pandemic, in which there is high mortality in individuals with underlying chronic inflammatory conditions. Vulnerable populations include the elderly and those with obesity, diabetes, and hypertension (1). The viral infection is characterized by an overproduction of various cytokines in severe cases, indicating that multiple inflammatory response systems are activated (2). The production of excess cytokines is thought to explain why some COVID-19 patients unexpectedly take a turn for the worse and do not survive (3). The mechanism underlying the cytokine storm is the subject of numerous hypotheses. We suggest that endotoxin, produced by Gram-negative gut bacteria, leaks out of a damaged gut and plays a role in the development of the cytokine storm.

It is now clear that the intestinal tract is likely to be a target for COVID-19 infection. Patients may experience diarrhea and vomiting during infection (4), and SARS-CoV-2 viral RNA has been detected in feces (5). The viral receptor angiotensin-converting enzyme-2 (ACE-2), required for viral entry into susceptible cells, has been found not only in the lung but also in the esophagus and the enterocytes of the ileum and colon (6). During the infection process, enterocytes are presumably infected, and the function of the intestinal membrane is likely compromised. One function of the intestine, as it relates to the cytokine storm, is that it serves as a barrier to prevent the leakage of microorganisms and their products into the bloodstream (7). The bacteria in the gut produce structurally diverse molecules called pathogen-activated molecular patterns (PAMPs), which can stimulate an immune response through Toll-like receptors (8). The PAMPs produced by Gram-negative bacteria include a glycolipid called lipopolysaccharide (LPS) or endotoxin. Endotoxin is part of the outer membrane of the bacterium and is shed during growth and bacterial cell death/lysis (9). It is detected in mouse and human feces (10, 11). Endotoxin stimulates the production of interleukin 6 (IL-6), IL-1, IL-8, tumor necrosis factor alpha (TNF-α), and gamma interferon (IFN-γ), cytokines that are also found in COVID-19 patients (12, 13). The inflammatory activity of endotoxin is structure dependent and varies by bacterial species and strain (14). For example, LPS of Bacteroides thetaiotaomicron, a prevalent fecal bacterium in the phylum *Bacteroidetes*, differs structurally from LPS of Escherichia coli and does not activate an inflammatory response (15). LPS produced by E. coli, a proteobacterium, is hexa-acylated, which accounts for its potent inflammatory activity, mediated via TLR4 (16). Endotoxin produced by gut bacteria is presumed to leak into the blood system and contribute to the development of an inflammatory response called metabolic endotoxemia (17). Further study of the gut microbiome is warranted to understand the variable inflammatory response to COVID-19 and whether intestinal lipopolysaccharide-producing Gram-negative bacteria are involved.

It is well known that diet modulates the gut microbiota and influences host health. The most abundant members of the bacterial communities in human feces belong to the phyla *Bacteroidetes* and *Firmicutes* (18). Under conditions of high dietary saturated fat, a second taxon of Gram-negative organisms, *Proteobacteria*, is detected in human feces in some individuals (19), while in other studies they were not (20). This may be related to the amount of fat or the vulnerability of the individual exposed. Increases in the abundances of *Proteobacteria* were reported in a study using humanized gnotobiotic mice fed a high-fat diet consisting of a mixture of saturated, monounsaturated, and polyunsaturated fats (21). In a meta-analysis of studies examining the effect of a high-fat diet of the mouse fecal microbiome, 15 of 25 murine studies showed that an increase in the *Firmicutes*-to-*Bacteroidetes* ratio was predictive of consumption of a high-fat diet (22). There were changes in three major clades identified: *Lachnospiraceae* and *Ruminococcaceae* within the *Firmicutes* and *Muribaculaceae* within the *Bacteroidetes*. Increases in abundances of fecal *Proteobacteria* are reported in studies examining the effect of a high-fat diet (23–25) in the mouse. The increase was accounted for by an increase in *Desulfovibrio* spp. (24, 25), an organism that produces a hexa-acylated LPS molecule, which is expected to have high inflammatory activity (26). Although the *Proteobacteria* in mouse feces are detected at low abundance compared to *Firmicutes* and *Bacteroidetes*, we hypothesize that the proinflammatory endotoxins produced by *Proteobacteria* may functionally contribute to an inflammatory response during a COVID-19 infection.

The observed effects of dietary fats on the gut microbiota, specifically the *Proteobacteria*, may be variable because *Proteobacteria* are not the dominant taxa in feces (and the large intestine) and therefore may be overlooked in results based on analysis of fecal samples. Results of early studies examining the microbiota of the human small intestine indicate that the relative abundance of *Proteobacteria* may be higher in the small intestine than in feces (27). For example, stomach, duodenal, jejunum, and stool samples were collected from 8 heathy subjects; *Proteobacteria* were not detected in the stool but were present in the small intestinal samples (27). Other studies of small intestinal microbiota relied on the use of subjects undergoing esophagogastroduodenoscopy (28), such as for gastroesophageal reflux disease (29). *Proteobacteria* were detected in the duodenal samples, but it is not clear what role the medical condition may have played in these individuals. It is, however, important to recognize that the abundance of bacteria is several orders of magnitude higher in the large intestine than in the small intestine. Clearly, these studies represent the first steps in the development of methods to understand microbial communities in the small intestine.

The detection of *Proteobacteria* in the small intestine raises the possibility that it is from this section of the intestine that endotoxin molecules with high proinflammatory activity translocate from the gut and contribute to the inflammatory response during a COVID-19 infection rather than from the endotoxin produced by the Gram-negative bacteria of the large intestine. In vulnerable populations, such as in obese individuals, it is known that postprandial endotoxemia is higher than in lean subjects (30) and that postprandial inflammation is higher in lean individuals after consuming cream compared to water (31). The increase in postprandial endotoxemia occurs within hours of meal ingestion, suggesting that absorption of endotoxin occurs after gastric emptying into the proximal small intestine (32). The increases in postprandial endotoxemia and inflammation may be due to chronic high intake of fat, which induces changes in the intestinal membrane permeability properties (33). Identifying the bacterial communities in the small intestines of lean versus obese individuals may lead to a better understanding of how intestinal bacteria might play a role in the cytokine storm that many COVID-19 patients experience. The use of a humanized mouse gut microbiota model to study the effect of diet and other exogenous factors might be an ideal way to understand the specific effects of different fatty acids on the gut microbiota. To study an endotoxin-mediated inflammatory response, developing model conditions with an animal species that is more sensitive to endotoxin, like humans, than mice are (34) could help unravel the role that different Gram-negative bacteria in the intestinal tract might play in inflammatory diseases.

There is an increasing number of studies examining the gut microbiota in at-risk populations for COVID-19 infection, such as those with diabetes and those who are obese. For example, a comparison of the fecal microbiomes of treatment-naive (TN) type 2 diabetic (T2D), prediabetic, and normal glucose-tolerant subjects (35) showed increases in multiple genera within the Gram-negative *Bacteroidetes* phylum only in the TN T2D patients. Interestingly, higher levels of Escherichia coli were detected in the pre-T2D subjects. E. coli, depending on strain, produces a highly proinflammatory endotoxin molecule (36). These results suggest that the Gram-negative communities in the intestinal tract of diabetic subjects may be enriched with bacterial strains/species that produce the most proinflammatory endotoxin molecules, which warrants further study. In obese individuals, there is lower diversity in the fecal bacterial communities and the *Firmicutes*-to-*Bacteroidetes* ratio is higher than in lean individuals (18), although there is some question as to whether an increased *Firmicutes*-to-*Bacteroidetes* ratio is a reproducible marker of obesity in humans (22).

Since blood levels of endotoxin are higher in obese individuals than in lean individuals (37), the results of human dietary intervention studies are of potential interest. In a 6-month randomized controlled-feeding trial using primarily soybean oil, a source of mono- and polyunsaturated fatty acids, increases in *Bacteroides* spp. were reported (38). There was no mention of an effect on *Proteobacteria*. In contrast, a small study consisting of healthy men fed a high-saturated-fat diet for 7 days reported an increase in *Betaproteobacteria* in a subset of individuals (19). Consumption of a high-fat diet (mixed fatty acids) by mice changes the fecal microbiome to raise the *Firmicutes*-to-*Bacteroidetes* ratio (39), while a diet high in saturated fats is associated with an increase in *Proteobacteria* (23–25). Results from murine studies (40, 41) indicate that the consumption of a diet rich in monounsaturated fatty acids, supplied as extra virgin olive oil, changes the fecal microbiome in a manner that is expected to lead to a reduction in endotoxins with proinflammatory activity. A decrease in the abundance of *Desulfovibrionaceae* was reported (40), and a decrease in the relative abundances of bacteria identified as aerobes and facultative anaerobes and bacteria likely to produce proinflammatory endotoxins was noted (41). These studies highlight the need for research to understand how dietary fats might modulate the types of bacteria that may produce highly inflammatory endotoxin molecules.

The reduction of gut *Proteobacteria* may be one way to reduce the level of inflammatory signals and thereby reduce the severity of a COVID-19 infection. In situations in which *Proteobacteria* in the gut are in high abundance, the leakage of proinflammatory endotoxin from the gut is hypothesized to add to the TLR4-mediated inflammation that the host develops in response to the viral infection. The acute lung injury that develops in SARS and in other conditions is mediated by host-derived oxidized phospholipid generated by NADPH oxidase-dependent production of reactive oxygen species as part of the immune response (42). Oxidized phospholipid is a potent stimulator of TLR4 (42). Interestingly, the lung pathology induced by influenza is reversed with a TLR4 antagonist, eritoran (43). It is thus of interest to understand whether high levels of circulating endotoxin in combination with host-derived TLR4 agonists (activator) are involved in triggering a more intense cytokine storm in vulnerable populations.

**FIG 1:**
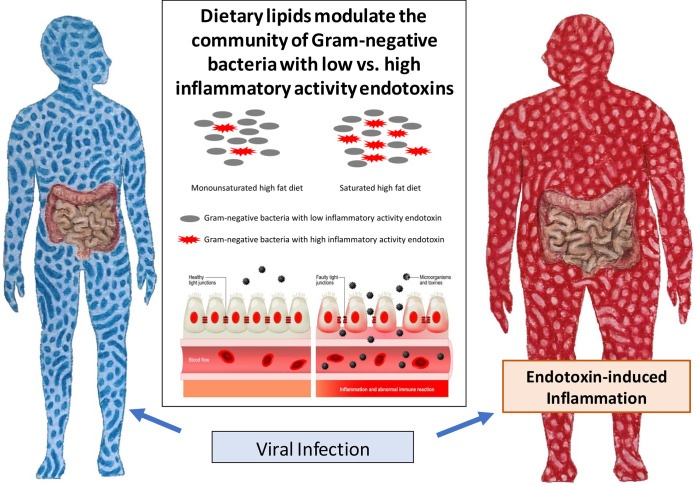
Intestinal permeability, altered gut microbiome, and fatty acid intake can raise the risk of endotoxin-induced inflammation. It is hypothesized that a viral infection in a patient with a high-risk condition exacerbates the inflammatory response.

In conclusion, we suggest that as we prepare to live with COVID-19, individuals with chronic inflammatory diseases should consider changing their diets before they are infected to attenuate the development of the most severe symptoms. A standard dietary intervention approach to mitigate chronic diseases is to decrease total fats, and while most agree that reducing the ratio of saturated fatty acids to monounsaturated fatty acids is beneficial, this is still debated (44). Here, we speculate, as depicted in Fig. 1, that shifting from a diet high in saturated fats to one with monounsaturated fats will reduce the numbers of those bacteria that produce the most inflammatory endotoxin molecules and thereby reduce the severity of the inflammatory response to a COVID-19 infection in vulnerable individuals, such as in obese individuals. Finally, although COVID-19 gains access to cells via the ACE-2 receptor, translocation of the virus from the gut to the systemic circulation should be considered if the intestinal membrane is compromised prior to the COVID-19 infection. The use of animal models to study the pathogenesis of COVID-19 will provide opportunities to more fully understand why this novel virus has devastating complications in some individuals but not in others.
